# Diagnostic Pitfalls and Challenges in Interpretation of Heart Transplantation Rejection in Endomyocardial Biopsies With Focus on our Experience

**DOI:** 10.5812/cardiovascmed.13986

**Published:** 2014-02-24

**Authors:** Kambiz Mozaffari, Hooman Bakhshandeh, Ahmad Amin, Nasim Naderi, Sepideh Taghavi, Zahra Ojaghi-Haghighi, Mahsa Abdollahi

**Affiliations:** 1 Rajaie Cardiovascular Medical and Research Center, Iran University of Medical Sciences, Tehran, IR Iran; 2 Cardiovascular Intervention Research Center, Rajaie Cardiovascular Medical and Research Center, Iran University of Medical Sciences, Tehran, IR Iran; 3 Echocardiography Research Center, Rajaie Cardiovascular Medical and Research Center, Iran University of Medical Sciences, Tehran, IR Iran

**Keywords:** Biopsy, Heart Transplantation, Quilty Effect, Graft Rejection

## Abstract

**Background:**

The current trend of heart transplantation in recent years has taken a quantum leap forward. We decided to look back at our experience in this center.

**Objectives:**

Here, we focus on the diagnostic pitfalls and challenges in these biopsies.

**Patients and Methods:**

Forty two patients based on the standard protocol of heart transplantation group, yielded 63 biopsy samples over a period of 33 months (April 2010 - December 2012). The mean age was 30.4 years (ranging from 16 to 58 years) with 51 males (81%) and 12 females (19%). All the patients were examined periodically and biopsy samples were taken from the right ventricular wall.

**Results:**

Rarely fewer than three pieces of myocardial samples were procured. Scar, adipose tissues and blood clots may be seen instead. Quilty effect (nodular endocardial lesions composed of inflammatory cell infiltrates) was seen in 8 cases (12.7%). Other findings not directly related to rejection including early ischemic injury, Quilty effect and post-transplant lymphoproliferative disorders (PTLD) were not encountered.

**Conclusions:**

Specimen inadequacy was not a major problem in our center. It poses a great limitation, because suboptimal specimens sometimes mislead the pathologist. Other findings especially Quilty effect were within the range defined for this finding.

## 1. Background

Considering the current trend of heart transplantation in recent years, which has obviously taken a quantum leap forward, we were prompted to look back at our experience to deal with future cases in a better way. Here, the main concerns are discussed from the pathologist’s vantage point in different aspects.

## 2. Objectives

Our aim was to seek the diagnostic pitfalls and challenges in the interpretation of transplant rejection in these biopsies, but we did not focus on the standard grading system for the pathologic diagnosis of transplant rejection in cardiac biopsies. (1-4).

## 3. Patients and Methods

Among a total of 42 patients with heart transplant referred for routine endomyocardial biopsy between April 2010 and December 2012, 63 biopsy samples were obtained for surveillance of the acute cellular rejection. All of the patients were scheduled for endomyocardial biopsy based on the International Society for Heart and Lung Transplantation (ISHLT) Guidelines for the Care of Heart Transplant Recipients (1). As most of the patients were referred to our center for the biopsy procedure from other hospitals, we did not have precise information regarding their first biopsy sampling or the interval between the biopsies; however, based on the routine protocol, in the first post-transplant year, the patients under monthly biopsies, unless there a suspicion of rejection prompting extra samplings.

The mean age of the patients was 30.4 years (ranging from 16 to 58 years) with 51 males (81%) and 12 females (19%). All the patients were examined periodically, and biopsy samples were taken from the right ventricular wall. Routinely, there is no need for a protracted hospital stay in this practice after the sampling procedure, and the patients can usually be discharged without any untoward complications. All the endomyocardial biopsies were taken to the histopathology laboratory where routine processes of embedding and staining are performed. Finally, the stained slides were reviewed and reports were issued by our pathologist. Here, we are not dealing with cases which showed various degrees of transplant rejection criteria introduced by the International Society for Heart and Lung Transplantation (ISHLT), but as was mentioned above, we are going to discuss the main concerns of the pathologist which should be observed as diagnostic pitfalls and challenges in the interpretation of transplant rejection in these biopsies. Table 1 depicts the most important morphological mimics of acute cellular rejection (5). The clinicians should bear in mind that multiple samples are needed from different right ventricle sites. In fact, a minimum of 3 or preferably 4 evaluable samples of myocardial tissue are recommended (2, 5).

**Table 1.** Histologic Differential Diagnosis for Acute Rejection

Item Number	Item
**1**	Perioperative ischemic injury
**2**	Catecholamine effect
**3**	Inflammatory changes in biopsy site
**4**	Infectious/viral myocarditis
**5**	Quilty effect
**6**	Post-transplant lymphoproliferative disorder (PTLD)
**7**	Recurrence of primary cardiac disease
**8**	Ischemic injury due to coronary artery diseases in the transplant

## 4. Results

**Figure 1.**
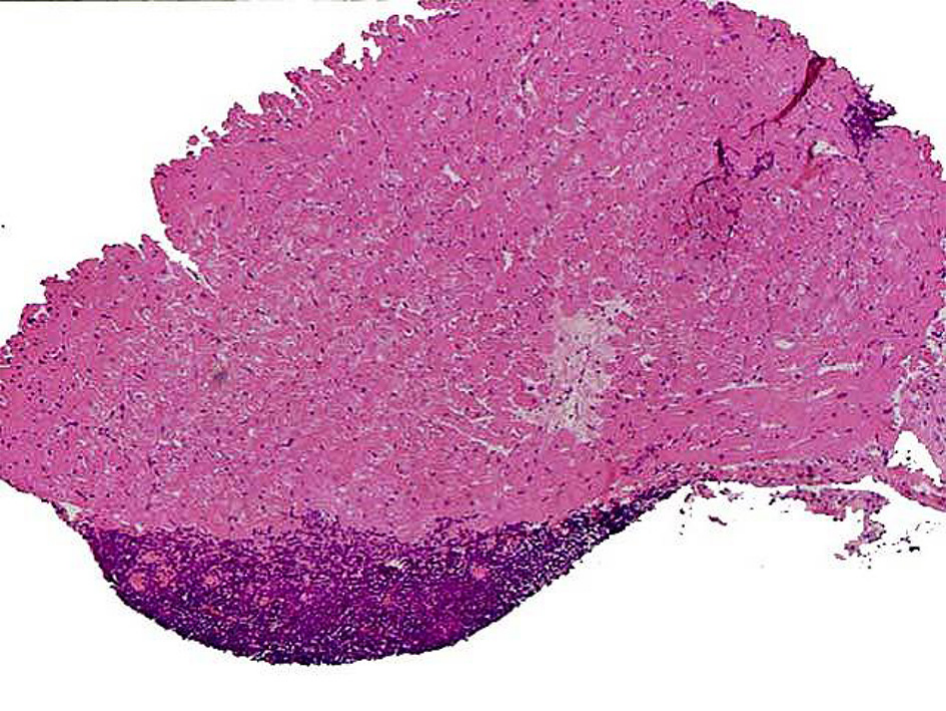
Quilty Effect, Nodular Endocardial Lesions Composed of Inflammatory Cell Infiltrates (H & E x 100)

On rare occasions, we noted that fewer than three pieces of acceptable myocardial tissue were procured. Scar or adipose tissues as well as blood clots were seen instead, thus compromising a satisfactory specimen collection and result interpretation. There were no pertinent findings attributable to viral myocarditis, both in our specimens and in the clinical presentations of our patients. Other biopsy findings which are not directly related to rejection, but may pose serious issues of concern, including early ischemic injury, Quilty effect, post-transplant lymphoproliferative disorders (PTLD). Fortunately, we had no such cases in our studies. Quilty effect was seen in eight cases (12.7%), which is within the range defined for this finding (Figure 1). 

## 5. Discussion

First and foremost, the issue of specimen inadequacy poses a great limitation in the proper interpretation of biopsies, in that suboptimal specimens sometimes mislead both the pathologist and the clinicians responsible for the patients. Another discouraged practice which was fortunately not seen here, thanks to the good understanding between the pathologist and clinicians, would occur if samples were divided into different containers, so as to be sent to different laboratories, in as much as it might result in less representative sampling, hence a suboptimal evaluation (3). Histopathologically, the first abnormal finding often misdiagnosed as acute cellular rejection is viral myocarditis. This is morphologically indistinguishable from rejection due to the fact that in both situations we encounter lymohocytic infiltration in myocardium, and if severe enough, the picture may be further aggravated by evidence of myocyte damage. Virological studies may help, and so do changes seen during histopathology examination in cases of Cytomegalic infections (intranuclear inclusion or peripheral halo in the cytoplasm), however, it is of paramount importance to consider the possibility of myocarditis, if no evidence of rejection is seen in the patient who has such inflammatory infiltrates, guiding the physicians to the diagnosis of myocarditis (3).

In another study with a larger number of patients (n = 814), CMV infection comprised 66% of viral infections (6). Early ischemic injury: tissue changes are caused by removal and implantation of the donor’s heart. Protracted hypotension in cases of poor graft function, or any untoward hemorrhage during the peri-operative period, are the culprits, not to mention the consequences of prolonged high-dose inotrope administration. The relevant morphological findings include formation of contraction bands or coagulative necrosis with vacuolization of myocyte fibers, and fat necrosis. With time, the process of healing would follow, and biopsies may contain mixed inflammatory infiltrates, such as neutrophils, lymphocytes, macrophages and eosinophils. Now, at this point, confusion with acute rejection may be likely. This type of ischemia-related injury is particularly more conspicuous in the healing phase, and is also a finding to be considered by the pathologist up to the first 6 weeks after performing the transplantation. Hence, clinical data is also of significance here.

In cases of ischemia, there is a relative lack of infiltrating inflammatory cells, versus a greater degree of myocyte damage seen as myocytolysis and vacuolization. We have not come across a straightforward case of ischemia-induced lesion, for at least two reasons. First, because most biopsies were taken from patients after the first six postoperative weeks when early ischemia-induced lesions are likely to be noted and second, because we have taken preventive measures to avoid prolonged ischemia in the transplant recipients (3-4). Quilty effect is by far the most frequent finding in our specimens and consists of nodular endocardial lesions composed of inflammatory cell infiltrates. Some authors have reported an incidence in post-transplant endomyocardial biopsies approaching 10% to 20%.

The infiltrates are either confined to the endocardial layer (Quilty A) or may extend deep into the myocardium underneath it (Quilty B). The latter is associated with myocyte damage. From the clinical point of view, it is not such an important issue, whether the Quilty lesion is confined to the endocardium or penetrates the myocardium with an invasive picture. This however, is significant for the pathologist and poses a big challenge in the interpretation, because if only part of the biopsy is viewed under the microscope with areas of myocyte invasion and damage, the pathologist is very likely to consider this area as a grade 1R or 2R (ISHLT) rejection. The safest policy here is to obtain different sections of tissue, that is, at least three different levels of tissue sections. In serial sections, the connection of myocyte injury to the overlying endocardium appears. Quilty effect’s association with acute rejection, is somewhat controversial, nonetheless, further studies in recent years have shown a greater possibility of developing rejection in patients who manifest this feature. Therefore, we add the note to our reports that close follow-up of the patient is advised. This lesion is believed to be distinct from acute rejection, needing no treatment with further immunosuppression.

Differentiation of Quilty lesion versus acute rejection is not usually a big problem when it is confined to the endocardium, as we mentioned previously; whereas, when it penetrates the underlying myocardium, viewing only a tangential cut through a biopsy sample may not show a genuine connection between the myocardial infiltration and the endocardial nodules, thus rendering differentiation from acute rejection a more cumbersome task. Once again, we emphasize the importance of obtaining additional deeper sections which can resolve this issue by revealing the extension of infiltration toward the endocardium. Factors which are in favor of Quilty effect include a dense infiltrate, presence of B cells and plasma cells, and a prominently fibrovascular background. Last but not least, immunohistochemical stains on the infiltrate demonstrate a combination of B and T cells and may prove helpful in this situation (1-10).

Other causes of infiltration in the myocardium include Infection and Post-Transplant Lymphoproliferative Disorders (PTLD). Both of these are regarded as significant causes of morbidity and mortality, albeit relatively rare in transplanted patients’ cardiac biopsies. A brief mention was made earlier regarding viral infections in these patients. Cytomegalovirus (CMV) and Toxoplasmosis, both may be accompanied by infiltrates rich in lymphocytes. Therefore, misinterpretation is likely, as the picture closely resembles that of acute cellular rejection, causing injudicious immunosuppression. Strict patient monitoring by means of viral Antibody titers and antiviral therapy could be the reason why we did not encounter such cases in this study. Adopting less aggressive immunosuppressive protocols, to diminish the chances of post-transplant neoplasms induced by viral infections, especially PTLD is also a matter which should always be taken into consideration. According to the authors, the latter is found in 2% of post-transplant patients (6). Given the fact that we had only a limited number of cases one would assume that this could well explain the lack of such findings in our series.

## References

[1] Hunt S, Burch M, Geetha B, Canter C, Chinnock R, Crespo-Leiro M (2010). The international society of heart and lung transplantation guidelines for the care of heart transplant recipients, task force3: long-term care of heart transplant recipients.. J Heart Lung Transplant..

[2] Cunningham KS, Veinot JP, Butany J (2006). An approach to endomyocardial biopsy interpretation.. J Clin Pathol..

[3] Stewart S, Winters GL, Fishbein MC, Tazelaar HD, Kobashigawa J, Abrams J (2005). Revision of the 1990 working formulation for the standardization of nomenclature in the diagnosis of heart rejection.. J Heart Lung Transplant..

[4] Fyfe B, Loh E, Winters GL, Couper GS, Kartashov AI, Schoen FJ (1996). Heart transplantation-associated perioperative ischemic myocardial injury. Morphological features and clinical significance.. Circulation..

[5] Mills SE, Carter D, Greenson JK, Reuter VE, Stoler MH (2010). Sternberg's Diagnostic Surgical Pathology..

[6] Silver MD, Gotlieb AI, Schoen FJ (2001). Cardiovascular Pathology..

[7] Kottke-Marchant K, Ratliff NB (1989). Endomyocardial lymphocytic infiltrates in cardiac transplant recipients. Incidence and characterization.. Arch Pathol Lab Med..

[8] Radio SJ, McManus BM, Winters GL, Kendall TJ, Wilson JE, Costanzo-Nordin MR (1991). Preferential endocardial residence of B-cells in the "Quilty effect" of human heart allografts: immunohistochemical distinction from rejection.. Mod Pathol..

[9] Joshi A, Masek MA, Brown BW, Weiss LM, Billingham ME (1995). “Quilty” revisited: A 10-year perspective.. Human pathology..

[10] Fishbein MC, Bell G, Lones MA, Czer LS, Miller JM, Harasty D (1994). Grade 2 cellular heart rejection: does it exist?. J Heart Lung Transplant..

